# Semi-synthetic vNAR libraries screened against therapeutic antibodies primarily deliver anti-idiotypic binders

**DOI:** 10.1038/s41598-017-10513-9

**Published:** 2017-08-29

**Authors:** Doreen Könning, Laura Rhiel, Martin Empting, Julius Grzeschik, Carolin Sellmann, Christian Schröter, Stefan Zielonka, Stephan Dickgießer, Thomas Pirzer, Desislava Yanakieva, Stefan Becker, Harald Kolmar

**Affiliations:** 1 Institute for Organic Chemistry and Biochemistry, Technische Universität Darmstadt, Alarich-Weiss-Strasse 4, D-64287 Darmstadt, Germany; 2 Merck Lab @ Technische Universität Darmstadt, Alarich-Weiss-Strasse 8, D64287 Darmstadt, Germany; 3 Antibody-Drug Conjugates and Targeted NBE Therapeutics, Merck KGaA, Frankfurter Straße 250, D-64293 Darmstadt, Germany; 4 Protein Engineering and Antibody Technologies, Merck KGaA, Frankfurter Straße 250, D-64293 Darmstadt, Germany; 5 Helmholtz-Institut für Pharmazeutische Forschung Saarland (HIPS), Department Drug Design and Optimization (DDOP), Campus E8.1, 66123 Saarbrücken, Germany

## Abstract

Anti-idiotypic binders which specifically recognize the variable region of monoclonal antibodies have proven to be robust tools for pharmacokinetic studies of antibody therapeutics and for the development of cancer vaccines. In the present investigation, we focused on the identification of anti-idiotypic, shark-derived IgNAR antibody variable domains (vNARs) targeting the therapeutic antibodies matuzumab and cetuximab for the purpose of developing specific capturing ligands. Using yeast surface display and semi-synthetic, CDR3-randomized libraries, we identified several highly specific binders targeting both therapeutic antibodies in their corresponding variable region, without applying any counter selections during screening. Importantly, anti-idiotypic vNAR binders were not cross-reactive towards cetuximab or matuzumab, respectively, and comprised good target recognition in the presence of human and mouse serum. When coupled to magnetic beads, anti-idiotypic vNAR variants could be used as efficient capturing tools. Moreover, a two-step procedure involving vNAR-functionalized beads was employed for the enrichment of potentially bispecific cetuximab × matuzumab antibody constructs. In conclusion, semi-synthetic and CDR3-randomized vNAR libraries in combination with yeast display enable the fast and facile identification of anti-idiotypic vNAR domains targeting monoclonal antibodies primarily in an anti-idiotypic manner.

## Introduction

Over the past decades, monoclonal antibodies have gained preeminent importance in the treatment of cancer as well as inflammatory and autoimmune diseases^1, 2^. With more than 30 antibody therapeutics being marketed and over 50 in late stage clinical trials, the market is growing more rapidly than ever^2–5^. This development, however, requires a reliable toolbox for the characterization and monitoring of these therapeutic entities as they progress into the clinic. While for some characterizations, such as the assessment of serum stability, the antigen can be employed as a capturing ligand, this approach may be limited when the antigen is not easily available, expensive or poses a safety threat^6–8^. The monitoring of a therapeutic antibody in patient serum can be particularly challenging and requires a molecule that exhibits excellent target recognition in the presence of excess IgG competitor molecules. In this case, anti-idiotypic antibodies which specifically recognize an idiotope located in the variable regions of a therapeutic antibody can be employed. Their high specificity for the unique antigenic determinants of the therapeutic antibody enable them to distinguish between their target and unrelated antibody variants. Such anti-idiotypic antibodies are of great importance in the assessment of the pharmacokinetic properties of a therapeutic antibody and can also be employed as a standard in the identification of anti-drug responses in order to analyze potential immunogenicity issues^9^. In addition to their utilization in various immunoassay formats, anti-idiotypic antibodies have been employed for the immunoaffinity enrichment of therapeutic antibodies, for mass spectrometric analyses^10, 11^, for the purification of bispecific antibodies^12^ and as cancer vaccines^13, 14^. With regard to their employment as cancer vaccines, anti-idiotypic antibodies which represent an ‘internal image’ of the actual antigen can be used for immunizations. The immune response that is elicited comprises anti-anti-idiotypic antibodies which in turn exhibit properties similar to those of the actual therapeutic antibody^15, 16^. This approach is especially feasible when self-antigens which are naturally non-immunogenic or even of a non-proteic nature are being addressed^13^.

**Figure 1:**
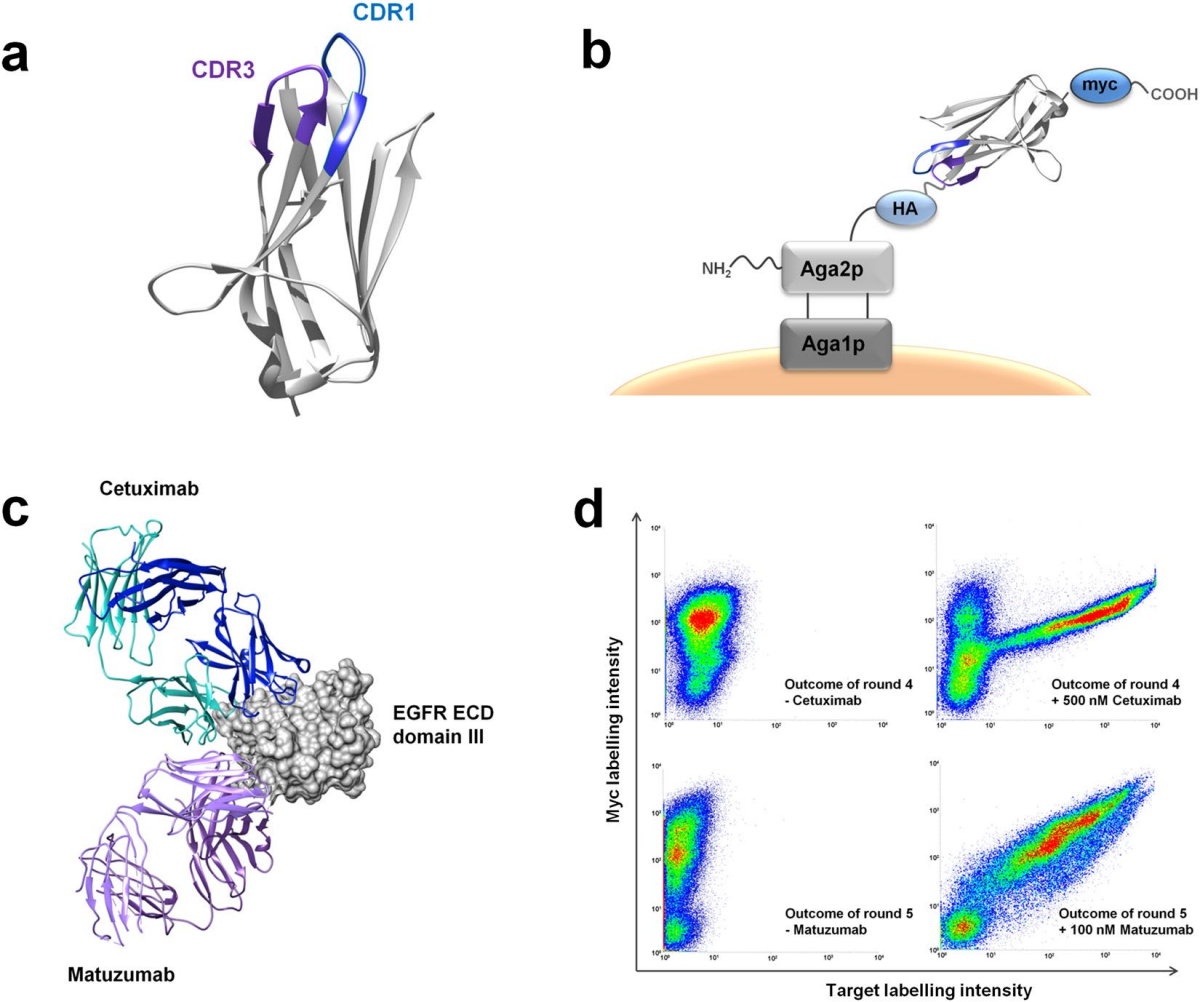
Screening of semi-synthetic, yeast-displayed vNAR libraries towards matuzumab and cetuximab. (**a**) Structural depiction of an IgNAR variable domain (vNAR). The CDR1 and CDR3 loops are highlighted in blue and purple, respectively. For the generation of semi-synthetic vNAR libraries in the scope of this work, only the CDR3 regions were randomized. The structure was modified from PDB identifier 4HGK using UCSF Chimera^49^. (**b**) Schematic depiction of the yeast surface display setup employed for the semi-synthetic vNAR libraries. Randomized vNAR domains are fused to the *C*-terminus of Aga2p. Full-length surface presentation is verified upon staining of the *C*-terminal myc tag. (**c**) Structural depiction of cetuximab and matuzumab Fab fragments targeting domain III of the extracellular portion of EGFR. It is apparent from the illustration, that both antibodies target neighbouring epitopes on EGFR. All structures were modified from PDB identifiers 1YY9 (cetuximab and EGFR) or 3C09 (matuzumab and EGFR domain III) using UCSF chimera^49^. (**d**) Dot plot diagrams depicting cells after the fourth or fifth sorting round towards cetuximab or matuzumab, respectively. Negative controls were incubated with secondary antibodies only (upper and lower left).

Many anti-idiotypic antibody and non-antibody molecules have been developed for this purpose. Examples include, among others, anti-idiotypic monobodies, scFvs, peptides or nanobodies^14, 17–21^. In the present investigation, we describe the identification of anti-idiotypic, shark-derived antibody domains targeting monoclonal antibodies. In comparison to conventional antibodies derived from mammals, cartilaginous fish (sharks, rays and skates) possess a unique form of heavy-chain only antibodies, termed immunoglobulin new antigen receptor or IgNAR^22^. Each heavy chain of this unique isotype possesses overall five constant domains followed by the variable domain (vNAR) that mediates antigen binding^22^. The absence of a light chain partner contributes to the high water solubility of vNAR domains since the hydrophobic V_H_-V_L_ interface of a conventional antibody is replaced by polar and charged amino acid residues^23–25^. Overall, vNAR domains possess two (CDR1 and CDR3) instead of three CDR loops, which is attributed to the structural depletion of a β-sheet^24^. However, the lack of a CDR2 binding site is at least in part compensated by the elongated CDR3 region that mediates the major part of antigen-binding (Fig. 1a). Additionally, their small size of approximately 11–12 kDa renders vNARs the smallest antibody-like antigen-binding domains known to date. Moreover, their small size and elongated CDR3 binding sites enable them to interact with structurally demanding, cleft-like epitopes which are often intractable for conventional antibodies^26–29^. The aforementioned characteristics as well as their high physicochemical stability render vNAR domains ideal for biomedical and biotechnological research and have led to the isolation of various vNAR candidates binding therapeutically relevant targets such as TNFα^30^, EpCAM^31^ or the malarial AMA protein^28^. In addition, it has been demonstrated that these domains can be engineered towards pH-sensitive target binding, opening up new avenues for the development of affinity ligands for tailor-made chromatographic applications^32^. Owing to these striking characteristics, we suggest that vNAR domains could be predisposed for the targeting of antibody variable regions. Their elongated CDR3 loops can potentially penetrate these regions and address recessed interspaces. Moreover, their high stability should enable their utilization in various immunoassay formats and under a variety of different conditions. Therefore, we wanted to investigate whether anti-idiotypic vNAR variants towards therapeutic antibodies could be isolated. In contrast to the common approaches described in literature, which have almost exclusively relied on phage display as high-throughput screening platform, we employed yeast surface display for the identification of anti-idiotypic binders (Fig. 1b)^33^. This bears the inherent advantage of simultaneous on-line and real-time analysis during fluorescence-activated cell sorting (FACS)^34^. In addition, the approach described in the present investigation relies on semi-synthetic instead of immune libraries, completely obviating the need for animal immunizations. This is an advantageous feature, especially when compared to the generation of camelid-derived heavy chain antibodies, which commonly involves the immunization of llamas or camelids^12, 14^.

In order to test the robustness of the presented approach, we chose cetuximab and matuzumab as target antibodies^35–38^. Both antibodies are directed towards the epidermal growth factor receptor (EGFR) and even engage neighbouring epitopes on domain III of its extracellular portion (Fig. 1c). While cetuximab has been approved by the FDA for the treatment of several cancer types, the clinical development of matuzumab has not been pursued^35^. In 2010, Hartmann and colleagues described the isolation of anti-idiotypic peptides targeting matuzumab and cetuximab from peptide phage libraries^15^. However, the selected library candidates were cross-reactive, comprising micromolar affinities for both target antibodies. Herein, we show that semi-synthetic, yeast-displayed vNAR libraries allow for the facile identification of highly specific and non-cross-reactive anti-idiotypic variants towards cetuximab and matuzumab. When immobilized on magnetic beads, these anti-idiotypic domains demonstrated their potential as capturing ligands in the presence of mouse serum as well for the immunoaffinity enrichment of bispecific cetuximab × matuzumab constructs.

## Results

### The isolation of anti-idiotypic vNARs from semi-synthetic yeast libraries does not require counter selections

**Figure 2:**
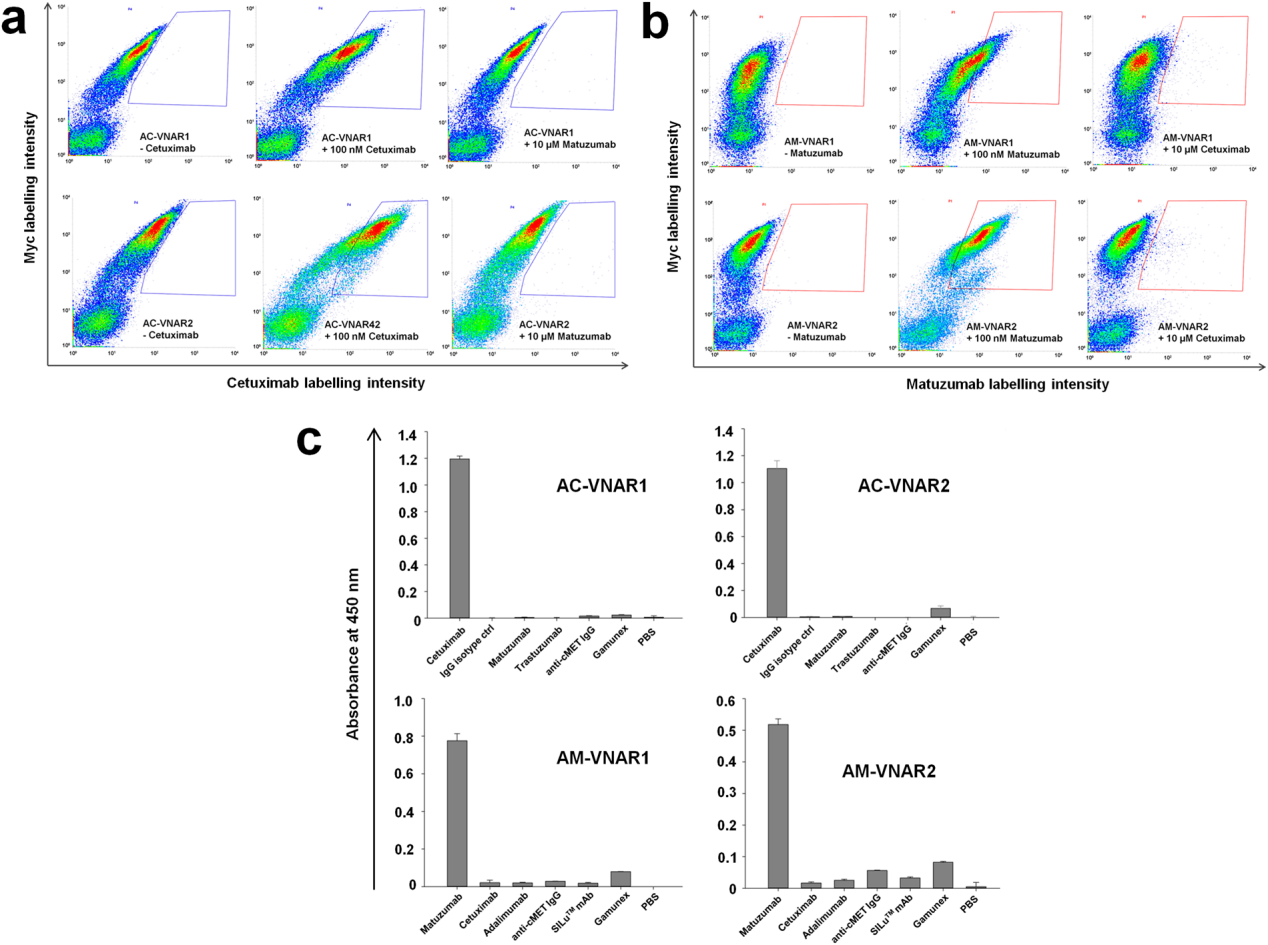
Single clones derived from rounds four and five towards cetuximab and matuzumab were tested in terms of target specificity on yeast and in an ELISA setup after recombinant expression. (**a**) Dot plot diagrams depicting yeast cells that display single clones AC-VNAR1 and AC-VNAR2 and were incubated with secondary labelling reagents (negative control), 100 nM cetuximab or 10 µM matuzumab. (**b**) Dot plot diagrams depicting yeast cells that present single clones AM-VNAR1 and AM-VNAR2 on their surface and were incubated with secondary labelling reagents (negative control), 100 nM matuzumab or 10 µM cetuximab. Full-length surface display was verified upon addressing the *C*-terminal myc tag. (**c**) Recombinantly expressed anti-idiotypic vNAR variants were coated into 96-well microtiter plates and incubated with cetuximab or matuzumab as well as other unrelated monoclonal antibodies and an IgG1 mixture (Gamunex®). Detection of bound antibodies was performed using a Fab-specific detection antibody conjugated to horseradish peroxidase. Experiments were performed in duplicates. Standard deviations are indicated in each diagram. In case of AC-VNAR1 and AM-VNAR2, 1000 nM of each antibody were employed. In case of AC-VNAR2 and AM-VNAR1, 500 nM of each antibody were utilized.

We generated overall four yeast-displayed vNAR libraries from naïve bamboo shark scaffolds as described previously^31^. The CDR3 binding site, which mediates the major part of the antibody-antigen interaction, was synthetically randomized using PCR and trimer-based oligonucleotide mixtures encoding for 12, 14, 16 or 18 amino acids, respectively, thereby extending the average length of 12 amino acids naturally found in the bamboo shark^31^. Randomized vNAR sequences were subjected to a second PCR, resulting in the assembly of the finalized inserts that comprised gap repair overhangs for homologous recombination in yeast. The generation of the four yeast libraries was performed using a high-yield transformation protocol^39^ and yielded a potential diversity of 3 × 10^9^ transformants altogether. After cultivation of the yeast libraries and induction of vNAR-expression in galactose-containing medium, surface presentation was detected upon fluorescent labelling of the *C*-terminal myc epitope tag (Fig. 1d). Overall, 35% of the analyzed cell population showed myc tag and, consequently, full-length expression of a vNAR domain. In order to analyze the library content in terms of binding to cetuximab, we performed target stainings with a 1 µM concentration of the antibody. Detection was performed using anti-human Fc-specific labelling antibodies. After four rounds of screening without the application of any counter selections, a significant portion of the yeast cell population showed strong binding to cetuximab (Fig. 1d). Subsequently, we analyzed the outcome of the fourth sorting round in terms of its sequence diversity. Overall, ten single clones were analyzed comprising only three different vNAR sequences. As one of the single clones possessed a CDR3 that was mainly composed of aromatic residues, potentially indicating non-specific interactions with the antibody target, we chose the two remaining anti-cetuximab vNARs AC-VNAR1 and AC-VNAR2 for further characterizations. Both variants were subsequently scrutinized in terms of anti-idiotypic binding towards cetuximab. As depicted in Fig. 2a, the single clones showed significant binding to 100 nM cetuximab, but no binding to 10 µM matuzumab. Also, no binding to other unrelated monoclonal antibodies was observable (Supplementary Figs 2 and 3). In addition, competition experiments involving an excess of a therapeutically utilized human IgG mixture (Gamunex®) were conducted. Both vNAR single clones were able to specifically target biotinylated cetuximab at a concentration of 10 nM and in the presence of 50 µM of Gamunex® (Supplementary Figs 2 and 3). In contrast to a positive control, that was performed without the addition of competitor, the binding capabilities of both clones were not impaired.

The screening procedure was repeated for the identification of vNAR library candidates that specifically engage matuzumab instead of cetuximab. Overall, five rounds of selection were performed in this case, resulting in a population that comprised binders with high affinity for matuzumab even in the presence of excess cetuximab (Fig. 1d and Supplementary Fig. S4). Out of overall ten single clones which were sent out for sequencing analyses, two unique sequences were obtained (Supplementary Fig. S1). These two single clones, termed AM-VNAR1 and AM-VNAR2, were then further characterized in terms of anti-idiotypic binding to matuzumab. As has been observed for the identified cetuximab binders, both clones showed distinct recognition of matuzumab but no binding to excess cetuximab (Fig. 2b) or other unrelated monoclonal antibodies (Supplementary Figs 5 and 6), indicating high specificity and anti-idiotypic binding. Competition experiments which involved the incubation of both single clones with 50 nM biotinylated matuzumab in the presence of 50 µM Gamunex® further emphasized their high specificity, as no competition and, therefore, no decrease in the binding signal could be observed (Supplementary Figs 5 and 6).

### Reformatted anti-idiotypic vNARs show high affinity and specificity

Single clones AC-VNAR1, AC-VNAR2, AM-VNAR1 and AM-VNAR2 were subcloned into a mammalian expression vector that enabled the expression of each vNAR as a fusion to a human IgG_1_ Fc region. Expi293F^TM^ cells were transiently transfected and the produced proteins were purified from the culture supernatants after five days using Protein A spin columns. The purity of all four constructs as Fc fusions was determined by analytical size exclusion chromatography and was higher than 93% (Supplementary Fig. S7). We then determined the affinities of the reformatted, bivalent vNAR-Fc constructs using Bio-Layer Interferometry (BLI). All four variants displayed affinities in the low nanomolar range, with AM-VNAR1 exhibiting a K_D_ value of 900 pM (Table 1, Supplementary Fig. S8). These bivalent affinities are most likely higher than those of the solitary vNAR domains, however, we propose, that they can potentially be engineered towards even higher affinities after conducting an incremental affinity maturation that involves randomization of the adjacent CDR1 loop (Fig. 1a)^31^. The specificity of all anti-idiotypic variants was further assessed in an ELISA format, as this type of immunoassay is the one most commonly used when characterizing a therapeutic antibody in the first place. Therefore, all four vNAR variants were coated onto microtiter plates at 4 °C over night. After a blocking step, incubations with cetuximab, matuzumab as well as several unrelated antibodies and Gamunex® were performed. Captured antibodies were detected using an anti-Fab-HRP conjugated antibody that interacts with the Fab region of the monoclonal antibodies but not with the vNAR-Fc fusion proteins. As depicted in Fig. 2c, specific binding to cetuximab is observable for single clones AC-VNAR1 and AC-VNAR2 (upper panel). Likewise, single clones AM-VNAR1 and AM-VNAR2 specifically engage matuzumab and exhibit only minor interactions with other, unrelated antibodies (Fig. 2c, lower panel). These minor interactions can most likely be attributed to the slightly increased percentage of aggregates observed for these vNAR variants during size exclusion chromatography analysis (Supplementary Fig. S7).

**Table 1:** Kinetic parameters of anti-idiotypic vNARs as Fc fusion proteins. Values were determined using Bio-Layer Interferometry.

vNAR variant	K_D_ (nM)	k_on_ (M^−1^ s^−1^)	k_dis_ (s^−1^)
AC-VNAR1	8.6 ± 0.1	2.6 × 10^5^ ± 3.2 × 10^3^	2.3 × 10^−3^ ± 1.1 × 10^−5^
AC-VNAR2	3.7 ± 0.098	1.7 × 10^6^ ± 4.2 × 10^1^	6.5 × 10^−3^ ± 5.5 × 10^−5^
AM-VNAR1	0.9 ± 0.027	4.0 × 10^4^ ± 9.5 × 10^1^	4.0 × 10^−5^ ± 1.1 × 10^−6^
AM-VNAR2	9.1 ± 0.071	7.2 × 10^4^ ± 4.4 × 10^2^	6.5 × 10^−4^ ± 3.2 × 10^−6^

### Anti-idiotypic vNARs selectively detect their target antibody in the presence of human serum

**Figure 3:**
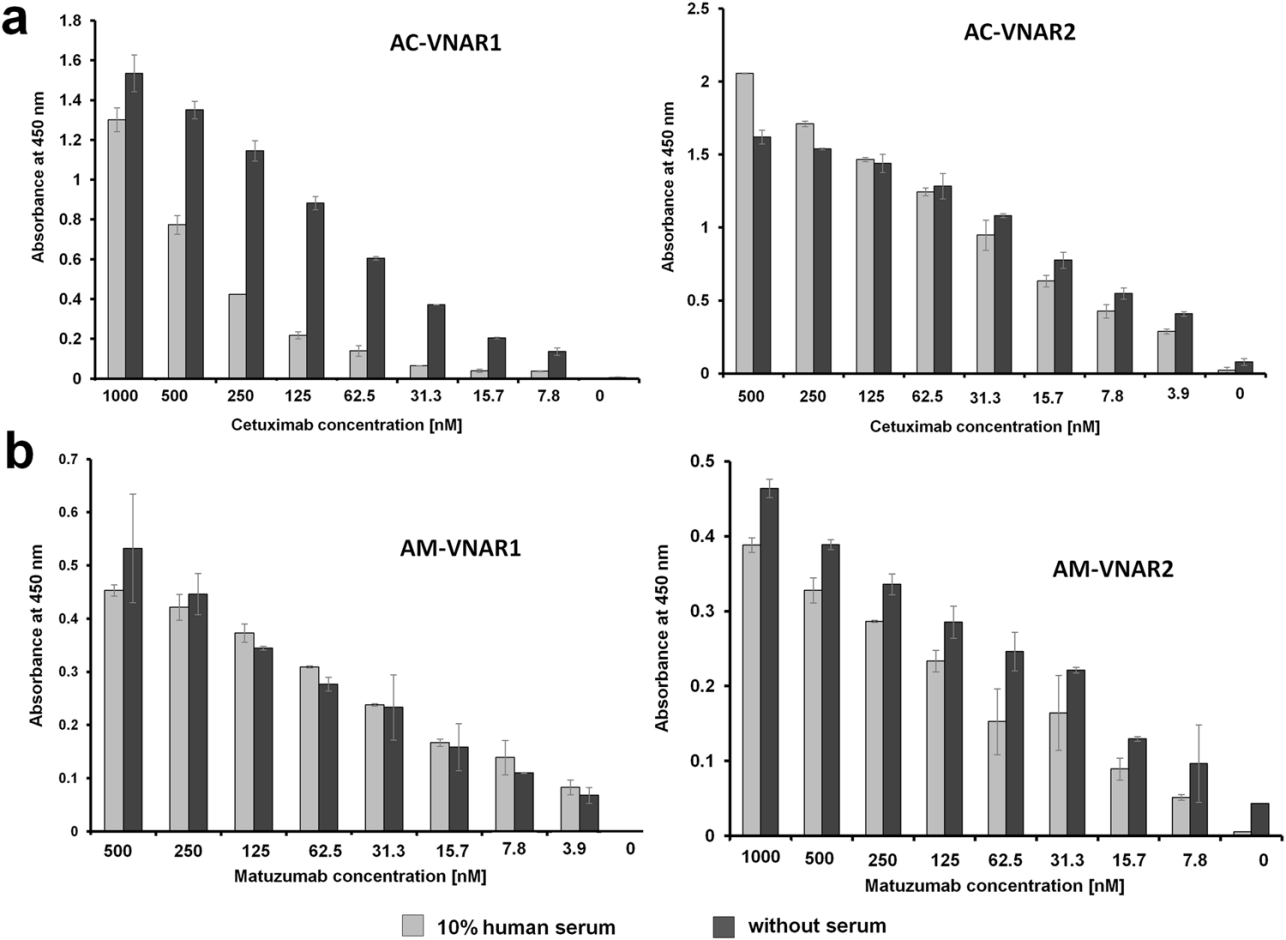
Results of the serum ELISA experiments encompassing anti-idiotypic vNARs. (**a**) Anti-idiotypic vNAR variants AC-VNAR1 and 2 were coated into 96-well microtiter plates. After a blocking step, wells were incubated with serial dilutions of biotinylated cetuximab in the presence (light gray) or absence (dark gray) of 10% human serum. Experiments were performed in duplicates, standard deviations are indicated in each diagram. (**b**) Anti-idiotypic vNAR variants AM-VNAR1 and 2 were coated into 96-well microtiter plates. After a blocking step, wells were incubated with serial dilutions of biotinylated matuzumab in the presence or absence of 10% human serum. Experiments were performed in duplicates, standard deviations are indicated in each diagram.

As serum stability assays as well as the quantification of a therapeutic antibody in patient serum are essential during the preclinical and clinical development of an antibody biologic, we next assessed whether anti-idiotypic vNARs were able to engage their biotinylated targets in the presence of 10% human serum. Therefore, decreasing concentrations of each biotinylated target antibody in the presence or absence of human serum were incubated with vNAR-coated wells. As depicted in Fig. 3a and b, only vNAR domains AC-VNAR2 and AM-VNAR1 were able to specifically recognize the biotinylated target antibody in the presence of human serum, whereas variants AC-VNAR1 and AM-VNAR2 showed decreased target binding when serum was present during the incubations. This indicates unspecific interactions with proteins present in human serum which can engage AC-VNAR1 and AM-VNAR2, hampering binding to cetuximab or matuzumab. As a consequence, only anti-idiotypic vNARs AC-VNAR2 and AM-VNAR1 were suited for further capturing assays in serum.

### Anti-idiotypic vNARs solely engage correctly assembled antibody paratopes

**Figure 4:**
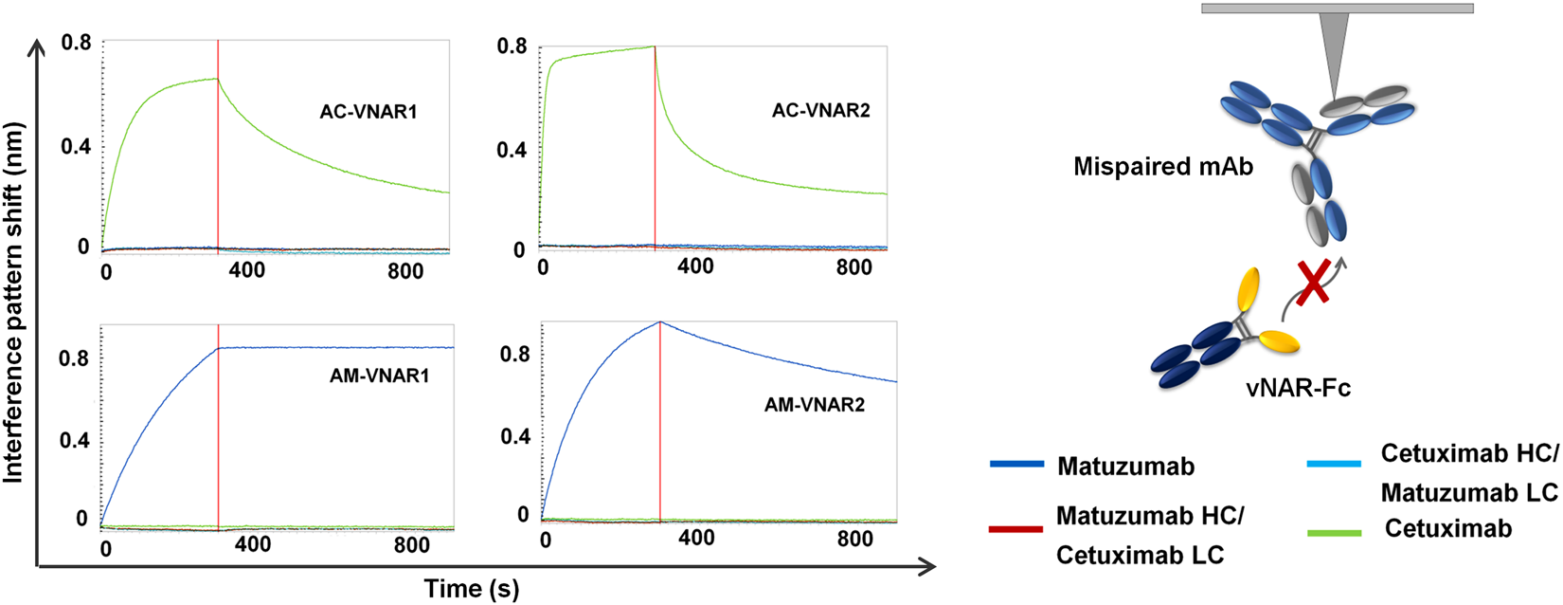
Assessment of paratope-specificity of anti-idiotypic vNAR-Fc variants using Bio-Layer Interferometry. Cetuximab, matuzumab or mispaired variants combining a matuzumab heavy chain with a cetuximab light chain and *vice versa* were immobilized on sensortips. After a baseline measurement in kinetics buffer, association of each anti-idiotypic vNAR-Fc was performed at a concentration of 100 nM. Subsequently, a dissociation step was carried out in kinetics buffer.

In order to investigate whether the idiotope that is being recognized by the anti-idiotypic vNAR variants on each target antibody is located on the heavy chain, the light chain or at the interface of both chains, we performed idiotype binding analyses. For this purpose, we co-transfected Expi293F^TM^ cells with plasmids encoding for the matuzumab heavy chain together with the cetuximab light chain (Matuz HC/Cetux LC) and *vice versa* (Cetux HC/Matuz LC). The culture supernatants were harvested after five days of production and purified using Protein A spin columns. Supplementary Fig. S9 depicts an SDS-PAGE of the mispaired variants after purification. Each lane contains protein bands which correspond to the heavy and light chain, verifying the correct assembly of these constructs. It is also apparent from the SDS-PAGE, that the cetuximab and matuzumab light chains differ slightly in size. Ultimately, matuzumab and cetuximab as well as the mispaired variants were immobilized on BLI sensor tips and subsequently transferred into wells containing the anti-idiotypic vNARs. As depicted in Fig. 4, only the complete antibody molecules consisting of correctly paired heavy and light chains were recognized. These results clearly demonstrate that the identified anti-idiotypic vNARs are paratope-specific for their corresponding target antibody and that both, the heavy and the light chain variable region are engaged in binding. It is interesting to note that solely paratope-specific binders rather than variants which engage the antibody constant regions were obtained, although no selection pressure was applied during the yeast display screenings^12^.

### Affinity capture of therapeutic antibodies from 5% mouse serum using magnetic beads

**Figure 5:**
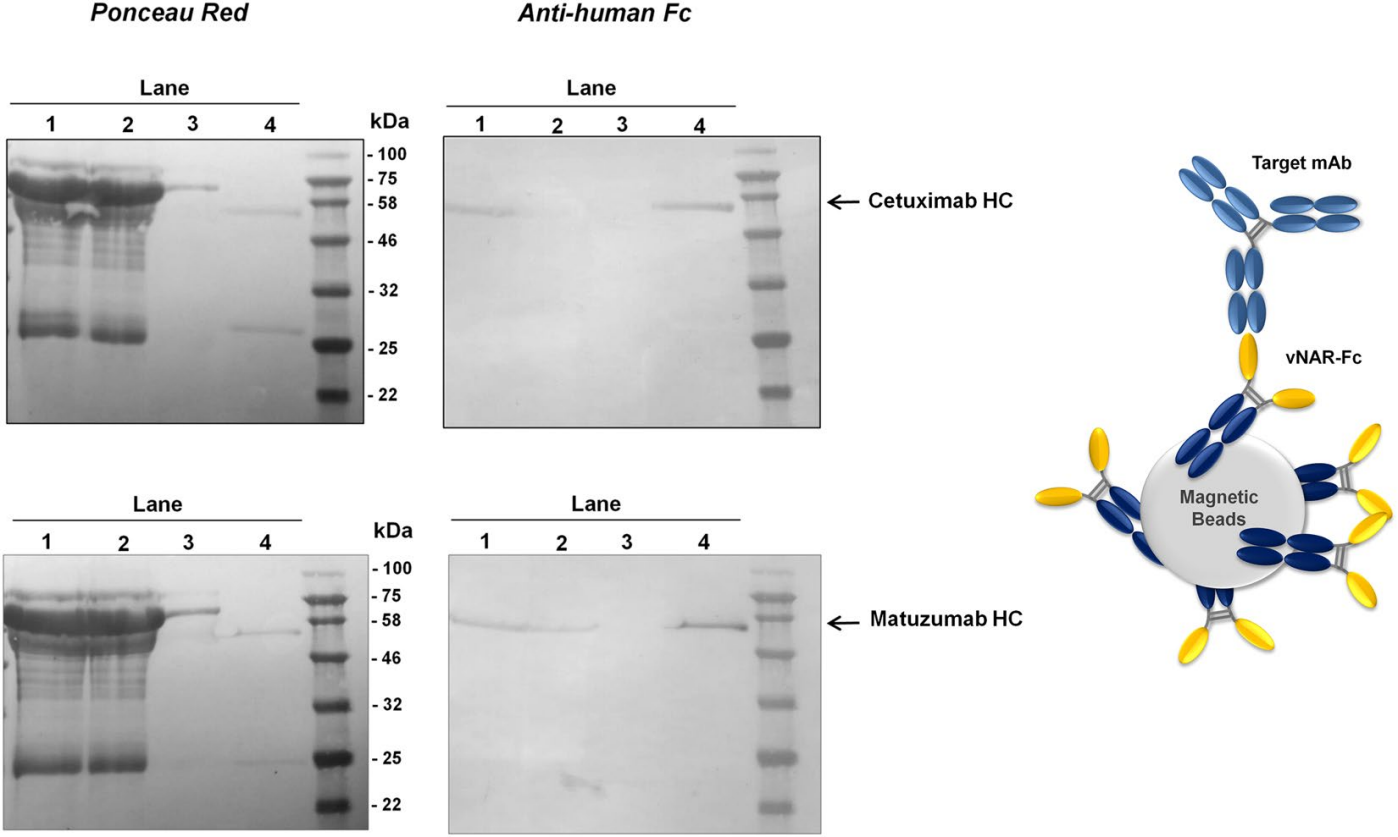
Anti-idiotypic vNAR-functionalized magnetic beads can selectively enrich cetuximab or matuzumab from 5% mouse serum. Streptavidin magnetic beads were coated with biotinylated AC-VNAR2 and AM-VNAR1, respectively. Subsequently, the functionalized beads were incubated with a mixture of 10 µg cetuximab or matuzumab (440 nM) in the presence of 5% mouse serum. The sample prior to application to the beads (lane 1), the flowthrough (lane 2), the first out of four washes (lane 3) and the eluate (lane 4) were subsequently applied to an SDS-PAGE and blotted onto a nitrocellulose membrane. The membrane was stained with Ponceau S in order to visualize the total amount of protein that was transferred from the SDS gel (upper and lower left). Afterwards, the membrane was destained and the human Fc region of cetuximab and matuzumab was detected using an anti-human Fc antibody that was conjugated to alkaline phosphatase (upper and lower right). It is observable, that the detected bands in the eluates correspond to enriched cetuximab or matuzumab heavy chains, respectively.

In order to investigate the capturing abilities of anti-idiotypic vNARs AC-VNAR2 and AM-VNAR1, we biotinylated both variants and incubated them with streptavidin-functionalized magnetic beads. After removal of unbound and unspecifically bound vNARs upon stripping of the beads with 0.1 M glycine buffer at pH 3, we incubated them with 10 µg (440 nM) of the corresponding therapeutic antibody in the presence of 5% mouse serum in PBS. Subsequently, the beads were washed several times with PBS and vNAR-bound antibodies were eluted using 0.1 M glycine buffer at pH 3. The purification procedure was illustrated on a western blot membrane that was stained with Ponceau S directly after the proteins were transferred from the SDS-PAGE (Fig. 5, upper and lower left). Ponceau S interacts with positively charged amine groups, thereby staining all the protein that has successfully been transferred to the western blot membrane. As depicted in Fig. 5, both capturing experiments encompassing AC-VNAR2 and AM-VNAR1 functionalized magnetic beads contain a high portion of serum proteins in both, the sample prior to application to the beads and in the flowthrough after bead incubation. The eluates of both experiments, however, comprise distinct bands corresponding to an IgG heavy and light chain with sizes of approximately 50 and 25 kDa (Fig. 5, upper and lower left). In order to prove that these bands correspond to the human therapeutic antibodies cetuximab and matuzumab instead of unspecific mouse IgG, we washed away the Ponceau S and specifically detected the human Fc part of both antibodies using an anti-human Fc alkaline phosphatase conjugate (Fig. 5, upper and lower right). Both, the samples prior to application to the beads and the eluates contain either cetuximab or matuzumab as indicated by a specific band that is visible after staining with the anti-human Fc conjugate. These results emphasize the potential of the identified anti-idiotypic vNARs as capturing agents for therapeutic antibodies in complex serum samples^40^. Applications for such vNAR-coated magnetic beads include, among others, the quantification of therapeutic antibodies in mass-spec based immuno-MRM assays^41^.

### Immunoaffinity enrichment of bispecific antibodies using anti-idiotypic vNARs

**Figure 6:**
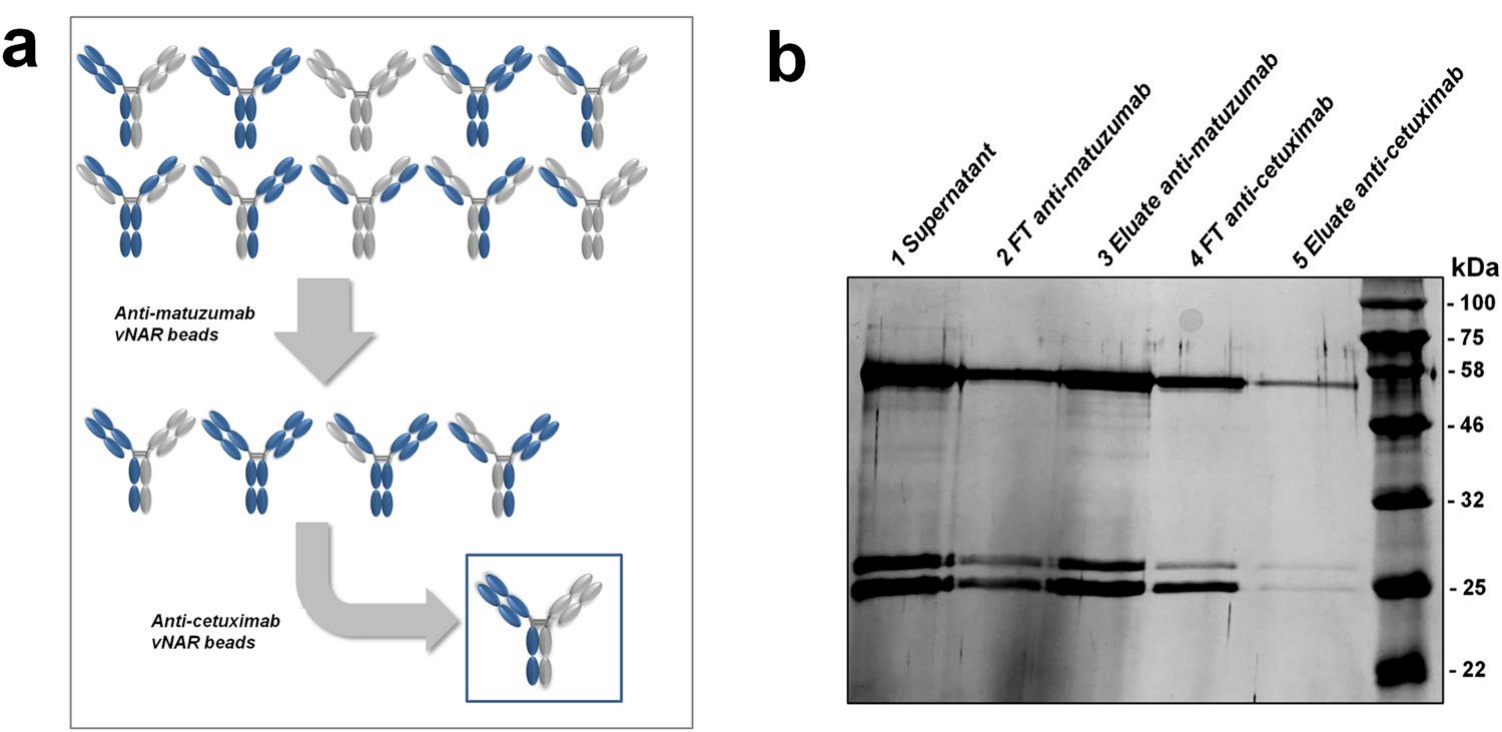
Immunoaffinity enrichment of bispecific cetuximab × matuzumab antibody constructs. (**a**) Schematic depiction of the enrichment procedure involving vNAR-functionalized magnetic beads. The heterogeneous antibody mixture that is obtained after co-transfection of heavy and light chains of matuzumab and cetuximab can potentially comprise ten different constructs. After the first purification step, only variants with an intact matuzumab paratope should be eluted, reducing the number of available molecules to four. After the last purification step, only the bispecific antibody variant is enriched since the anti-idiotypic vNARs presented in this investigation exclusively interact with correctly assembled paratopes. (**b**) The two-step purification process was illustrated on a SDS-PAGE. Since there is a slight difference in weight between the cetuximab and matuzumab light chains, the intensity of each light chain band can be utilized as a guideline during purification. The last lane containing the final eluate exhibits equal intensities for both light chains, underpinning a successful enrichment. For reasons of clarity, the supernatant as well as the flowthrough after incubation with the anti-matuzumab beads were purified using Protein A magnetic beads.

Analogous to the work recently published by Godar and colleagues, we wanted to investigate whether anti-idiotypic vNARs were able to identify bispecific antibodies from a heterogeneous mixture^12^. In this manner, a two-step purification procedure involving the AM-VNAR1 and AC-VNAR2 vNAR-functionalized magnetic beads should conducted. To generate a mixture of antibodies comprising various combinations of heavy and light chains, we co-transfected the heavy and light chains of cetuximab and matuzumab in HEK293 cells and harvested the supernatant after five days of production. A fraction of the concentrated supernatant was diluted in PBS (1:1) before it was added to the AM-VNAR1-coated magnetic beads. The co-transfection of all four chains results in the generation of overall 10 different antibody constructs (Fig. 6a)^12^. Since the identified anti-idiotypic vNARs do not interact with mispaired heavy and light chains, the first purification step should reduce the number of available antibody variants to four (Fig. 6a). In that manner, only antibodies comprising an intact matuzumab paratope should interact with the beads. Subsequently, the eluate from the AM-VNAR1-functionalized beads was buffer-exchanged to PBS and added to AC-VNAR2-coated magnetic beads. During this second purification step, only the bispecific antibody comprising an intact cetuximab and an intact matuzumab paratope should interact with the matrix. The purification process was monitored on an SDS-PAGE (Fig. 6b). Due to the slight difference in weight of the matuzumab and cetuximab light chains, the intensity of each light chain band can be utilized as a guideline during the step-wise purification. Whereas the first eluate after incubation with AM-VNAR1-coated beads contains a higher amount of matuzumab light chains, the second eluate reveals the presence of equal amounts of both light chains. This indicates that the two-step capturing procedure succeeded and that only the bispecific antibody variant is obtained after the final elution step.

## Discussion

In the present investigation, we added shark-derived IgNAR variable domains to the list of binders that can be engineered towards anti-idiotypic binding of monoclonal antibodies recognizing discontinuous epitopes. Our approach includes CDR3-randomized, semi-synthetic libraries and therefore omits the need for animal immunizations. In addition, our libraries are thoroughly sampled using yeast surface display. To our knowledge, this is the first time that yeast display was utilized as a high-throughput platform for the identification of anti-idiotypic binders^42^. Subsequently, we could demonstrate that reformatted and bivalent vNAR single clones, comprising bivalent affinities in the pico- to nanomolar range for matuzumab or cetuximab, were highly specific for their cognate antigen, even in the presence of human or mouse serum. Despite the fact that no selection pressure was applied during fluorescence-activated cell sorting, all binders solely engaged the antigen-binding region of the respective target monoclonal antibody, while no binders against the constant regions were obtained. Moreover, binding required the correct pairing of cetuximab or matuzumab heavy and light chain. Mispaired variants combining the cetuximab heavy with a matuzumab light chain and *vice versa* were not recognized. Although no co-crystal structures of these anti-idiotypic vNARs in complex with their target antibodies are available, we propose that these domains might be predisposed for the engagement of antibody variable regions. A possible explanation for the isolation of vNAR variants which almost exclusively interact with the antibody variable regions could be the hydrophobic nature of the paratope. It is well-known that antibodies often comprise aromatic residues such as Phe, Tyr and Trp in their CDR binding sites^43^. Such residues could represent favorable interaction sites for vNAR domains. Indeed, the CDR3 loops of the isolated anti-cetuximab and anti-matuzumab vNARs comprise a relatively high number of aromatic histidine residues, which can be attributed to the initial library design (Supplementary Fig. S1). These histidine residues could interact with aromatic CDR residues of monoclonal antibodies via π-π stacking or cationic π-interactions^44, 45^, delivering an explanation for the observation that only vNAR domains targeting the paratope rather than the antibody constant regions are identified. However, it needs to be scrutinized more meticulously whether these effects are also observed when non-histidine-enriched libraries are being employed.

**Figure 7:**
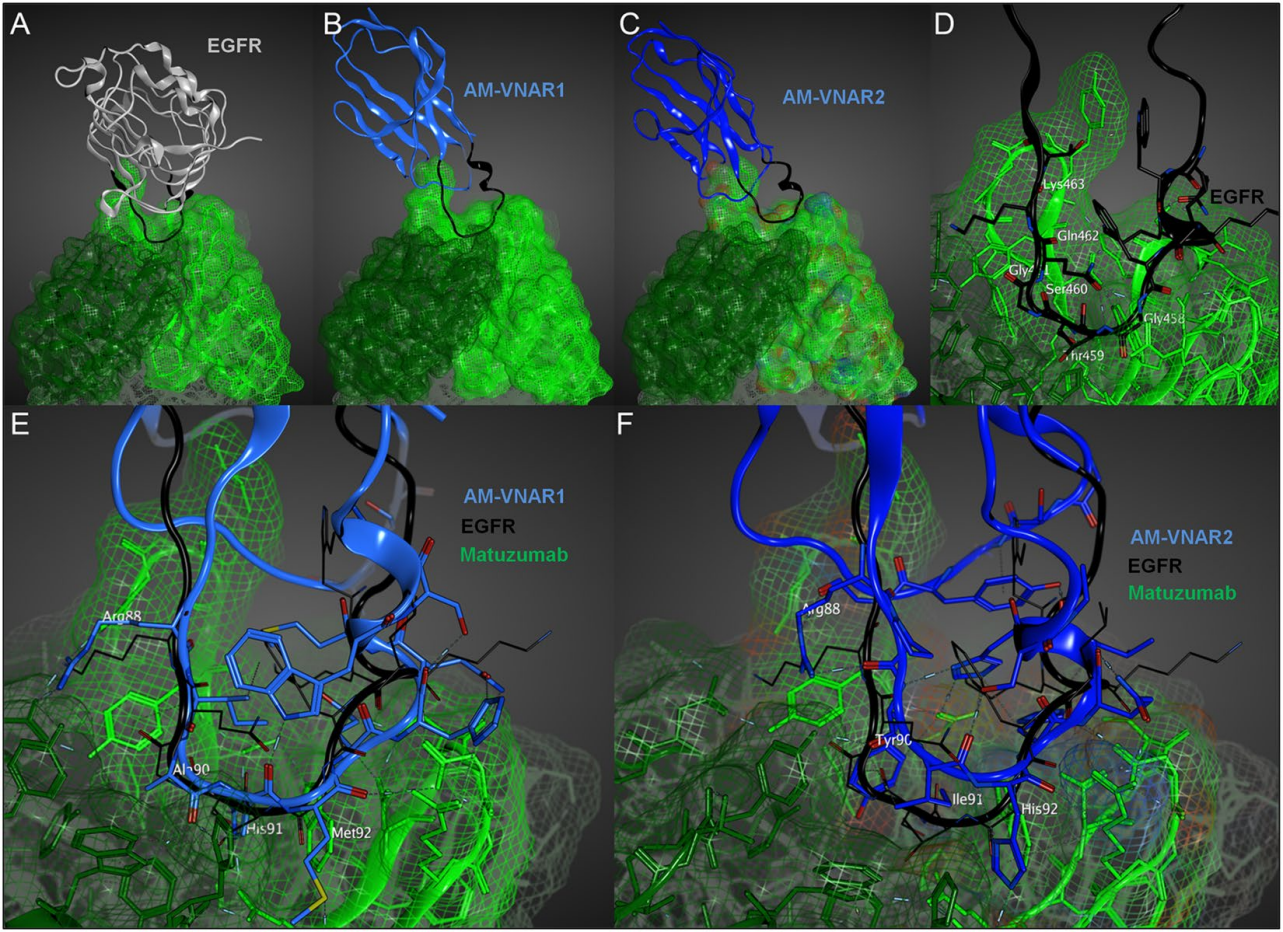
*In silico* modelling of binding poses for anti-matuzumab vNARs AM-VNAR1 and AM-VNAR2. (**A**) Matuzumab (green) in complex with domain III of the extracellular portion of EGFR (grey). The corresponding loop region that mediates the interaction with matuzumab is depicted in black. Structures were modelled from PDB identifier 3C09. (**B**) and (**C**) Modelled binding poses for AM-VNAR1 and AM-VNAR2 (blue), respectively, in complex with matuzumab. The CDR3 binding site is depicted in black. VNAR X-ray structures were selected based on sequence similarity and sequence coverage (PDB identifier 2I26). (**D**) Close-up view of the matuzumab-engaging loop of EGFR domain III. (**E**) and (**F**) Structural overlay and close-up view of AM-VNAR1 (E) and AM-VNAR2 (F, depicted in blue) as well as the respective EGFR loop (black) in complex with matuzumab. All homology models were built using Molecular Operating Environment (MOE, Chemical Computing Group).

It is also tempting to speculate that anti-idiotypic vNARs are structurally able to engage interspaces at the interaction site of heavy and light chains of a monoclonal antibody, which could be attributed to their elongated CDR3 binding sites^29^. Although antibody paratopes exhibit a rather flat structural architecture, vNARs could be able to penetrate this region with high specificity, as indicated by the results presented in this investigation and also previous results published by Simmons and coworkers^42^. Since our investigations focused on monoclonal antibody targets that engage a conformational rather than a linear epitope on their respective antigen, it is fairly difficult to identify sequence similarities between EGFR and the anti-idiotypic vNARs presented in this work. We exemplarily proposed a structural model for the anti-matuzumab vNARs using molecular modelling in order to explain why these domains almost exclusively recognize the antibody paratope. As depicted in Fig. 7, the CDR3 binding sites of AM-VNAR1 and AM-VNAR2 potentially adopt a conformation similar to that of the matuzumab-engaging loop of EGFR domain III (Fig. 7A,B and C). Notably, a loop-helix motif proposed for the CDR3 binding site of both vNAR domains seems to correlate with a related structure identified in the EGFR backbone (Fig. 7D). In case of AM-VNAR1, several amino acid residues of the CDR3 binding site overlap with residues of EGFR that possess similar interaction abilities. These residues include Lys463, Gly461 and Ser460 of EGFR which overlap with residues Arg88, Ala90 and His91 of AM-VNAR1, respectively (Fig. 7E). For AM-VNAR2, the modelled binding poses were not as conclusive as for AM-VNAR1. However, the observed similarity between amino acids residues Lys463 of EGFR and Arg88 were likewise observable (Fig. 7F). Additionally, the hydrogen bond donor as well as acceptor properties displayed by Ser460 of EGFR could functionally be mimicked by residues Tyr90 or His92 of AM-VNAR2. These *in silico* modelling experiments could at least provide a partial explanation for the observation that our semi-synthetic libraries exclusively yield anti-idiotypic binders.

Anti-idiotypic binders can generally be employed for a wide range of different applications, ranging from the characterization of therapeutic antibodies to the profiling of auto-antibodies in patients suffering from autoimmune diseases^21^. Anti-idiotypic binders which represent an ‘internal image’ of the antigen of the respective target antibody have also been employed as cancer vaccines, thereby circumventing the problem of immune tolerance that is associated with most tumour-associated antigens^13, 14^. Anti-idiotypic entities can bypass this problem upon eliciting an immune response that is directed towards their own paratope. These anti-anti-idiotypic antibodies in turn often exhibit similar properties to the actual therapeutic antibody that has been utilized for the generation of anti-idiotypic candidates. Thus, not only antibodies towards tumour-associated proteins but also towards non-proteic antigens such as carbohydrates have efficiently been utilized for their development and, towards this end, for the immunization and vaccination of animals^13, 46, 47^.

The identification of anti-idiotypic binders which are able to specifically target cetuximab and matuzumab has already been described by Hartmann and colleagues in 2010^15^. Their experiments initially encompassed phage display selections of randomized peptide libraries that should engage either cetuximab or matuzumab. However, the identified peptide candidates revealed cross-reactivity and were not able to distinguish between the two therapeutic antibodies. Importantly, no cross-reactivity was observed for the anti-idiotypic vNARs identified in our investigation. Interestingly, the peptides identified from both phage screening campaigns conducted by Hartmann and colleagues possessed two common amino acid motifs, namely KTL and YPLG^15^. When looking at the sequences from our anti-idiotypic vNARs, we were able to identify a motif similar to YPLG (YGLG, YVLG and YALG) in the CDR1 binding sites (Supplementary Fig. S1). The tyrosine residue has been introduced manually prior to randomization of the CDR3 loop and library generation since bamboo shark-derived vNAR sequences naturally comprise a cysteine residue at this position^31^. Upon mutating this residue to tyrosine and excluding cysteine residues during the CDR3-randomization process, we wanted to circumvent the formation of any disulfide bonds between CDR1 and CDR3^31^. Although these YXLG motifs were present in both sets of anti-idiotypic binders, they do not seem to affect their specificity as no cross-reactivity for the matuzumab binders towards cetuximab and *vice versa* was observable. However, since no crystal structures of anti-idiotypic vNARs in complex with their cognate target antibody are available, we can only speculate about the general contribution of the non-randomized CDR1 binding site to the actual protein-protein interaction. We also identified anti-idiotypic vNARs targeting other monoclonal antibodies that do not naturally engage EGFR (data not shown) and those sequences likewise comprised a YXLG sequence in CDR1. These antibodies were not able to interact with either cetuximab or matuzumab, leading to the conclusion that this amino acid motif is not associated with target specificity in our case. Likewise, Simmons and coworkers identified anti-idiotypic vNARs with no obvious sequence homology to the three amino acid minimal motif recognized by their target antibody, suggesting the formation of a similar but discontinuous motif caused by contributions from CDR1^42^. Notably, our anti-cetuximab vNARs also did not comprise any sequence homology to the anti-cetuximab peptides published by Riemer and colleagues^48^.

We further demonstrated that anti-idiotypic vNARs might be suitable for the immunoaffinity enrichment of cetuximab × matuzumab bispecific antibodies. Analogous to the approach recently published by Godar and coworkers, anti-idiotypic entities which are specific for intact antibody paratopes can be utilized for the capture of bispecific antibodies from complex mixtures^12^. Indeed, we could show that after a two-step purification procedure using anti-idiotypic vNAR-functionalized magnetic beads, equal intensities for the cetuximab and matuzumab light chains were observable on the corresponding SDS-PAGE. As only small amounts of functionalized beads were utilized for the proof-of-concept purification, the amount of the bispecific construct that was obtained after the second elution step was quite low. In addition, both parts of the cetuximab × matuzumab bispecific antibody engage EGFR, which makes it rather difficult to precisely prove the bispecific nature of the purified construct. Nevertheless, our anti-idiotypic binders verifiably only engage correctly assembled heavy/light chain combinations and a similar light chain segregation pattern during purification has also been observed by Godar and colleagues, underpinning a successful enrichment.

In conclusion, highly specific anti-idiotypic vNAR variants can easily be identified from semi-synthetic libraries. They were characterized and produced in less than three months, enabling a fast and customizable process for each individual target antibody. This generic yeast display and library screening approach obviates animal immunization and delivers binding proteins for monoclonal antibodies with ramifications for applications in antibody analytics and purification.

## Materials and Methods

### Yeast strains, media and reagents

The *Saccharomyces cerevisiae* strain EBY100 was utilized for yeast surface display^33^. YPD medium was composed of 20 g/L tryptone, 20 g/L dextrose and 10 g/L yeast extract. SD-CAA medium was prepared using 6.8 g/L yeast nitrogen base without amino acids but supplemented with ammonium sulphate, 5 g/L Bacto Casamino Acids, 20 g/L dextrose, 8.6 g/L NaH_2_PO_4_ × H_2_O, and 5.4 g/L Na_2_HPO_4_. SG-CAA medium was prepared similarly except for the substitution of dextrose sugar with galactose. Phosphate-buffered saline (PBS) contained 8.1 g/L NaCl, 0.75 g/L KCl, 1.13 g/L Na_2_HPO_4_ and 0.27 g/L KH_2_PO_4_. Cetuximab and matuzumab antibodies were kindly provided by Merck.

### Yeast library construction

The PCR-amplified vNAR products from overall three non-immunized bamboo sharks served as template for a consecutive three-step PCR. It is important to notice that the vNAR templates comprised a cysteine to tyrosine mutation in CDR1, which was introduced during the first PCR reaction. A detailed scheme can be found in a previously published paper from our group^31^. During the second PCR, randomization of either 12, 14, 16 or 18 amino acids in the CDR3 regions was carried out using a pre-assembled trinucleotide mixture (EllaBiotech), wherein each amino acid-encoding trimer except the one for cysteine was utilized. The trimer mixtures were biased towards the introduction of a higher percentage of histidine. As such, 2–4 histidine incorporations per loop (depending on its length) should be incorporated, allowing for the selection of potentially pH-sensitive variants^32^. During the third PCR, homologous sequences for gap repair cloning in yeast with the linearized pCT vector were attached^33^. The respective PCR products were purified using the Wizard® SV Gel and PCR Clean-up System (Promega) according to the manufacturer’s protocol. The pCT plasmid used for gap repair cloning and surface presentation of vNAR variants was digested with the restriction enzymes *Nhe*I-HF and *Bam*HI-HF and purified using the Wizard® SV Gel and PCR Clean-up System (Promega). For each electroporation reaction, 1.5 µg of digested pCT plasmid were combined with 4.5 µg of insert. Overall, 10 transformation reactions per library were performed. Dilution plating was carried out in order to determine the library size after three days. Yeast cells were transferred to SD-CAA medium and grown for two days at 30 °C^39^.

### Yeast library screening

Prior to library screenings, plasmid DNA from positive clones was isolated and sent out for sequence analysis. Library screening for the isolation of anti-idiotypic vNAR variants was performed on a BD Influx^TM^ cell sorter and analyzed *via* BD FACS^TM^ Sortware v1.0. For each of the two first sorting rounds, 8 × 10^8^ cells were labelled with either 1 µM cetuximab or matuzumab. The detection of vNAR-displaying cells exhibiting target binding was generally carried out using an anti-c-myc-biotin antibody (mouse, monoclonal, Miltenyi Biotec), an anti-human IgG antibody (Fc gamma-specific, goat, monoclonal, diluted 1:100, affymetrix eBioscience) or an anti-human Fc specific Fab fragment that was conjugated to Alexa Fluor® 488 (diluted 1:200 in PBS, goat, Jackson ImmunoResearch) as well as either streptavidin conjugated to allophycocyanin (diluted 1:50 in PBS pH 7.4, affymetrix eBioscience) or R-phycoerythrin (diluted 1:100 in PBS, Life Technologies). Incubation steps were performed at room temperature for 1 h (cetuximab and matuzumab) or 20 minutes on ice (secondary labelling reagents), respectively. The following rounds were performed upon staining at least ten times the number of yeast cells sorted in the previous round in order to ensure adequate library coverage. Secondary labelling reagents were alternated in order to circumvent the enrichment of vNARs interacting with these reagents.

### Reformatting and recombinant expression of anti-idiotypic vNARs as Fc fusions

Reformatting of the identified anti-idiotypic vNARs was accomplished upon genetically fusing them to an IgG1 Fc region encoded in the mammalian expression vector pEXPR-IBA42. VNAR variants were connected with the Fc region via a H20 hinge linker. Notably, the Fc sequence contained an Asn297Ala mutation in order to prevent glycosylation of the resulting fusion protein. Ligated constructs were sequence-verified and expressed in Expi293F^TM^ cells subsequent to ExpiFectamine^TM^-mediated transient transfection according to the manufacturer’s instructions (Life Technologies). Culture supernatants were harvested, sterile-filtered (0.22 µm Stericup filter, EMD Millipore) and purified using ProSep^®^ Protein A columns (EMD Millipore). Buffer exchange was performed with 10 kDa Amicon Ultra filter units (EMD Millipore). Analytical size exclusion chromatography was carried out using a TSKgel SuperSW3000 column (4.6 × 300 mm, Tosoh) at a flow rate of 0.35 mL/min.

### Specificity and serum ELISA assays

For specificity ELISA assays, anti-idiotypic vNARs were coated onto Nunc MaxiSorp® plates at 1–3 µg/mL at 4 °C over night. Subsequently, plates were blocked using 3% bovine serum albumin (BSA) in PBS for at least 2 h at room temperature. Plates were washed thrice with PBS 0.1% Tween-20. Incubations using cetuximab, matuzumab or other unrelated antibodies and the IgG mixture Gamunex® (Grifols Deutschland GmbH) were carried out for 1 h at room temperature and upon employing 500 nM or 1000 nM of the respective antibodies in PBS 1% BSA. In case of AM-VNAR1 and AM-VNAR2, incubations were conducted with biotinylated antibodies. Biotinylation was carried out using a two-fold molar excess of EZ-Link Sulfo-NHS-LC-Biotin (Thermo Fisher Scientific) for 30 minutes at room temperature. Biotinylated antibodies were subsequently purified using Protein Desalting Spin Columns (MWCO 3 kDa, Thermo Fisher Scientific). Unrelated antibodies were either kindly provided by Merck KGaA, Darmstadt (Adalimumab, anti-cMET IgG and isotype control), purchased from Sigma-Aldrich (SILu^TM^ Lite SigmaMab) or recombinantly expressed in HEK293 cells in-house (Trastuzumab). After washing the plates thrice with PBS 0.1% Tween-20, captured antibodies were labelled using an anti-Fab specific antibody conjugated to horseradish peroxidase (HRP, diluted 1:5000 in PBS 1% BSA, Sigma-Aldrich) or an Extravidin horseradish peroxidase conjugate (diluted 1:5000 in PBS 1% BSA, Sigma-Aldrich). Incubations were performed at room temperature for 30 minutes. After washing the plates thrice with PBS 0.1% Tween-20, a 1:1 mixture of TMB One Solution (Promega) and water was added to each well. Ultimately, reactions were stopped using 0.2 M HCl and plates were read on a Tecan Infinite F200 plate reader.

Serum ELISA assays were performed likewise. Biotinylated cetuximab and matuzumab were added to vNAR-Fc coated plates at a concentration of 500 nM or 1000 nM in the presence of either 10% human serum (Merck Millipore) or PBS 1% BSA and were serially diluted by a factor of 2. Captured antibodies were detected using Extravidin-HRP.

### Capturing assays using vNAR-functionalized magnetic beads

VNAR-Fc fusion proteins AC-VNAR2 and AM-VNAR1 were biotinylated using a four-fold molar excess of EZ-Link Sulfo-NHS-LC-Biotin (Thermo Fisher Scientific) for 30 minutes at room temperature. Biotinylated antibodies were subsequently purified using Protein Desalting Spin Columns (MWCO 3 kDa, Thermo Fisher Scientific). Streptavidin magnetic beads were purchased from GE Healthcare (Streptavidin Mag Sepharose) and functionalized with biotinylated vNARs according to the manufacturer’s instructions. After coupling, magnetic beads were stripped using 0.1 M glycine buffer at pH 3. Overall, about 77 µg of AC-VNAR2-Fc and 60 µg of AM-VNAR1-Fc were coupled to 50 µL of bead slurry. For capturing experiments, a 440 nM solution (10 µg of each antibody in total) of either cetuximab or matuzumab with 5% mouse serum (kindly provided by Merck) in PBS was added to AC-VNAR2- or AM-VNAR1-coated beads, respectively. After incubation at 4 °C for 30–60 minutes, the beads were washed at least 4 times using 500 µL PBS. Elution was carried out using 50 µL 0.1 M glycine buffer at pH 3. The eluate was subsequently transferred into a new tube containing 1 M Tris buffer at pH 9.

After running the collected samples on a 15% SDS-PAGE, proteins were blotted onto a nitrocellulose membrane (Amersham^TM^ Protran^TM^ 0.45 µm NC, GE Healthcare Life Science). Blotted membranes were subsequently stained using Ponceau S solution (0.1% (w/v), 5% acetic acid; Sigma-Aldrich). Destaining was conducted using PBS 0.1% Tween-20. After a blocking step with 3% milk powder for at least 2 hours, the membrane was washed several times with PBS and was subsequently incubated with an anti-human Fc specific antibody conjugated to alkaline phosphatase (diluted 1:5000 in PBS with 1.5% milk powder, Sigma-Aldrich). Afterwards, the membrane was consecutively washed with PBS, water and alkaline phosphatase buffer (100 mM NaCl, 50 mM MgCl_2_ × 6 H_2_O, 100 mM Tris, pH 9.1). Bands were developed using 12 µl of a 75 mg/mL solution of NBT in 70% DMF and 75 µL of a 50 mg/mL solution of BCIP in 100% DMF.

### Production and capturing of bispecific cetuximab × matuzumab antibodies

Initially, HEK293 cells were transfected with four plasmids encoding for the matuzumab heavy and light chains and cetuximab heavy and light chains. After five days of production, the culture supernatant was harvested and concentrated using a 10 kDa Amicon Ultra filter unit (EMD Millipore). The concentrated supernatant was then subjected to incubations using AM-VNAR1-functionalized magnetic beads according to the procedure described for the capturing assays in mouse serum. The eluates after the elution step were buffer-exchanged against PBS using Protein Desalting Spin colums (Thermo Fisher Scientific) and then applied to AC-VNAR2-coated beads. Incubation and elution was carried out as described above. The final eluates were subsequently concentrated using 10 kDa Amicon Ultra filter unit (EMD Millipore). After separation of the collected samples on a 15% SDS-PAGE, a silver staining was performed.

### Bio-Layer Interferometry measurements

Determination of binding kinetics was performed on an Octet^®^ RED96 system (fortéBio) at 30 °C, orbital sensor agitation at 1000 rpm and in 200 µL volume. Anti-Fab-CH1 or streptavidin sensor tips were pre-wet in PBS for 10 minutes. Subsequently, matuzumab or cetuximab were loaded onto the tips at a concentration of 5–10 µg/mL for 300 s. Afterwards, a baseline measurement was done in kinetics buffer (PBS, 0.1% BSA, 0.02% Tween-20) for 120 s. Association was performed using decreasing concentrations of anti-idiotypic vNAR-Fc fusion proteins for 300 s. Dissociation was performed in kinetics buffer for 600 s (AC-VNARs) or 900 s (AM-VNARs). Sensorgrams were fitted using a 1:1 Langmuir binding model (Analysis Software version 9.1) after subtraction of control curve date (without ligand).

Idiotope binnings were carried out upon immobilizing cetuximab, matuzumab, matuzumab HC/cetuximab LC and cetuximab HC/matuzumab LC on streptavidin sensortips for 300 s. After a baseline measurement in kinetics buffer for 120 s, association using 100 nM of each anti-idiotypic vNAR was carried out for 300 s, followed by a dissociation step in kinetics buffer for 600 s.

### *In silico* modelling of binding poses

Binding poses of AM-VNAR1 and AM-VNAR2 were modelled based on the co-crystal structure of matuzumab and EGFR (PDB-ID: 3C09). To this end, a vNAR X-ray structure was selected based on sequence similarity and sequence coverage. PDB entry 2I26 was identified as a suitable scaffold for generating homology models of both matuzumab binders (AM-VNAR1: similarity 69%, gaps 0%; AM-VNAR2: similarity 67%, gaps 4%; data accessed via NCBI protein BLAST). Homology models were built using Molecular Operating Environment (MOE, Chemical Computing Group: *Molecular Operating Environment* (*MOE*), 2015; Chemical Computing Group Inc., 1010 Sherbooke St. West, Suite #910, Montreal, QC, Canada, H3A 2R7, 2017.). The standard settings and AMBER10:EHT as force field with the R-field solvation model were employed. The main interacting loop of EGFR (aa447–468) showed significant structural similarity to the CDR3 loops observed in our homology models of AM-VNAR1 and AM-VNAR2. Hence, matuzumab binders were aligned to this EGFR region. Resulting poses were energy-minimized after repacking of CDR3 amino acid side chains in the matuzumab environment. Both steps were conducted as standard operations in MOE using AMBER10:EHT and the R-field solvation model.

## Electronic supplementary material

Supplementary Information
