# Supplementary material for: Semi-synthetic vNAR libraries screened against therapeutic antibodies primarily deliver anti-idiotypic binders
Source: Sci Rep. 2017 Aug 29;7:9676. doi: 10.1038/s41598-017-10513-9 (PMC5575089; doi:10.1038/s41598-017-10513-9)
Supplement: Supplementary file 1 — Supplementary Information [file 41598_2017_10513_MOESM1_ESM.pdf]

Supplemental

Semi-synthetic vNAR libraries screened against therapeutic antibodies primarily deliver anti-idiotypic binders

Doreen Könning[1], Laura Rhiel[2], Martin Empting[4], Julius Grzeschik[1], Carolin Sellmann[3], Christian A. Schröter[2], Stefan Zielonka[3], Stephan Dickgießer[2], Thomas Pirzer[1], Desislava Yanakieva[1], Stefan Becker[3], Harald Kolmar[1*]

[1]Institute for Organic Chemistry and Biochemistry, Technische Universität Darmstadt, Alarich-Weiss-Strasse 4, D-64287 Darmstadt, Germany

[2]Antibody Drug Conjugates and Targeted NBE Therapeutics, Merck KGaA, Frankfurter Straße 250, D-64293 Darmstadt, Germany

[3]Protein Engineering and Antibody Technologies, Merck KGaA, Frankfurter Straße 250, D-64293 Darmstadt, Germany

[4]Helmholtz-Institut für Pharmazeutische Forschung Saarland (HIPS), Department Drug Design and Optimization (DDOP), Campus E8.1, 66123 Saarbrücken

[*]To whom correspondence should be addressed:

Harald Kolmar: Institute for Organic Chemistry and Biochemistry, Technische Universität Darmstadt, Alarich-Weiss-Straße 4, D-64287 Darmstadt, Germany

Email: Kolmar@Biochemie-TUD.de

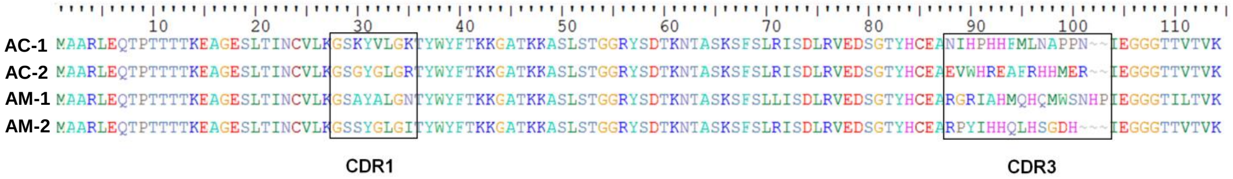

Figure S1: Sequence alignment of anti-ID vNAR single clones AC-VNAR1, AC-VNAR2, AM-VNAR1 and AM-VNAR2. The CDR1 and CDR3 binding sites are highlighted in black rectangles.

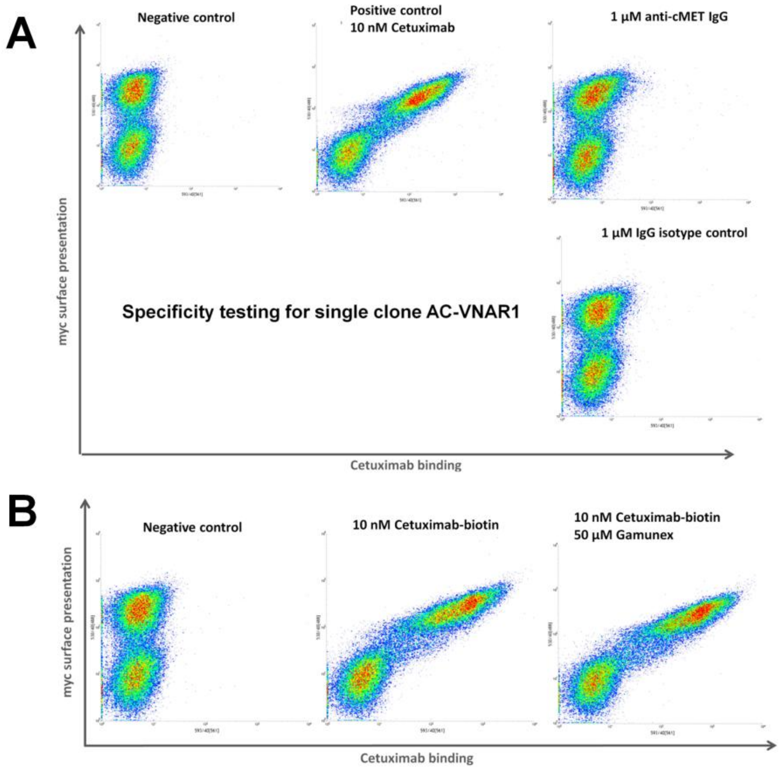

Figure S2: Specificity of yeast-displayed anti-cetuximab vNAR AC-VNAR1. A) Cells were incubated either with secondary labeling reagents only (negative control), with 10 nM cetuximab (positive control) or with 1 µM of unrelated control IgGs. Immunofluorescence stainings were performed upon employing an anti-c-myc-FITC antibody and an anti-human Fc-specific antibody labeled with PE. B) Yeast cells were incubated with secondary labeling reagents only (negative), 10 nM biotinylated cetuximab (positive control) or 10 nM biotinylated cetuximab pre-incubated with 50 µM of Gamunex® (20 min on ice). Immunofluorescence stainings were carried out upon employing an anti-c-myc-FITC antibody and streptavidin-R-phycoerythrin (SPE).

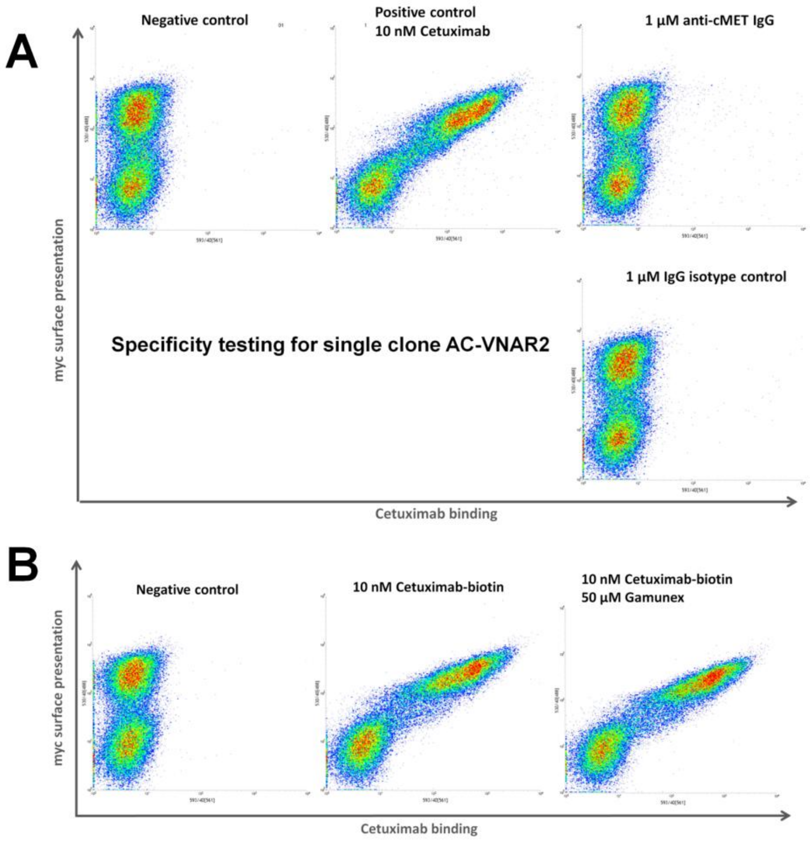

Figure S3: Specificity of yeast-displayed anti-cetuximab vNAR AC-VNAR2. A) Cells were incubated either with secondary labeling reagents only (negative control), with 10 nM cetuximab (positive control) or with 1 µM of unrelated control IgGs. Immunofluorescence stainings were performed upon employing an anti-c-myc-FITC antibody and an anti-human Fc-specific antibody labeled with PE. B) Yeast cells were incubated with secondary labeling reagents only (negative), 10 nM biotinylated cetuximab (positive control) or 10 nM biotinylated cetuximab pre-incubated with 50 µM of Gamunex® (20 min on ice). Immunofluorescence stainings were carried out upon employing an anti-c-myc-FITC antibody and streptavidin-R-phycoerythrin (SPE).

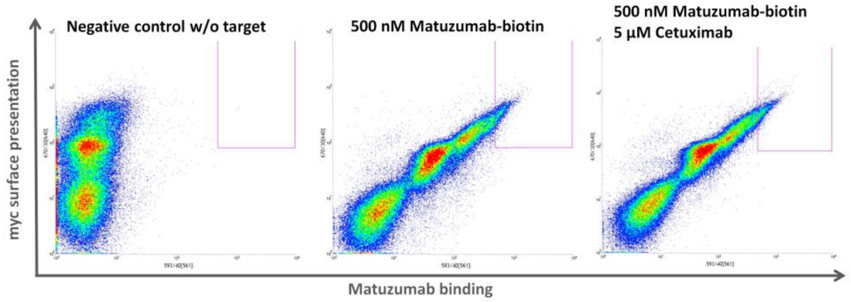

Figure S4: Specificity of yeast-displayed anti-matuzumab vNARs after screening round 4. Cells were incubated either with secondary labeling reagents only (negative control), with 500 nM biotinylated matuzumab (positive control) or with 500 nM biotinylated matuzumab in the presence of 5 µM cetuximab.

Immunofluorescence stainings were performed upon employing an anti-c-myc antibody, an anti-mouse-APC antibody an streptavidin-R-phycoerythrin (SPE).

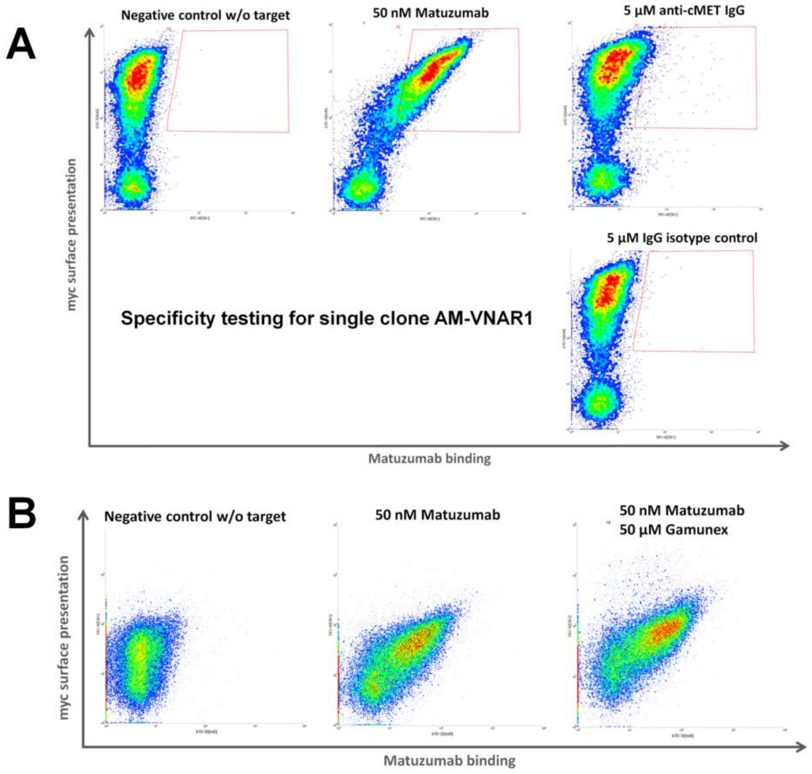

Figure S5: Specificity of yeast-displayed anti-matuzumab vNAR AM-VNAR1. A) Cells were incubated either with secondary labeling reagents only (negative control), with 50 nM matuzumab (positive control) or with 5 µM of unrelated control IgGs. Immunofluorescence stainings were performed upon employing an anti-c-myc antibody, an anti-mouse-allophycocyanin antibody and an anti-human Fc-specific antibody labeled with PE. B) Yeast cells were incubated with secondary labeling reagents only (negative), 50 nM biotinylated matuzumab (positive control) or 50 nM biotinylated matuzumab pre-incubated with 50 µM of Gamunex® (20 min on ice). Immunofluorescence stainings were carried out upon employing an anti-c-myc antibody, an anti-mouse-PE antibody and streptavidin-allophycocyanin (SAPC).

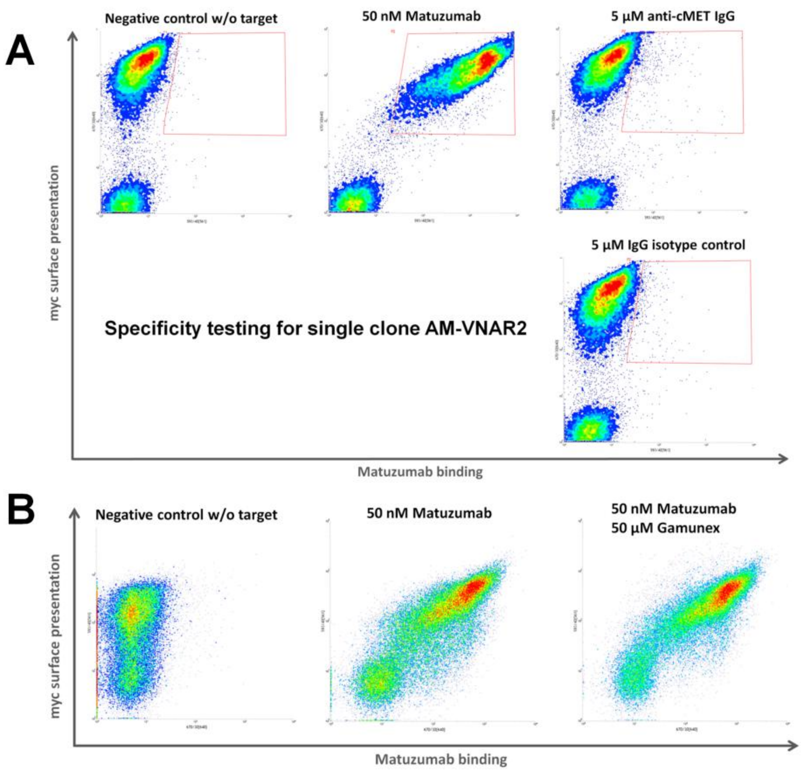

Figure S6: Specificity of yeast-displayed anti-matuzumab vNAR AM-VNAR2. A) Cells were incubated either with secondary labeling reagents only (negative control), with 50 nM matuzumab (positive control) or with 5 µM of unrelated control IgGs. Immunofluorescence stainings were performed upon employing an anti-c-myc antibody, an anti-mouse-allophycocyanin antibody and an anti-human Fc-specific antibody labeled with PE. B) Yeast cells were incubated with secondary labeling reagents only (negative), 50 nM biotinylated matuzumab (positive control) or 50 nM biotinylated matuzumab pre-incubated with 50 µM of Gamunex® (20 min on ice). Immunofluorescence stainings were carried out upon employing an anti-c-myc antibody, an anti-mouse-PE antibody and streptavidin-allophycocyanin (SAPC).

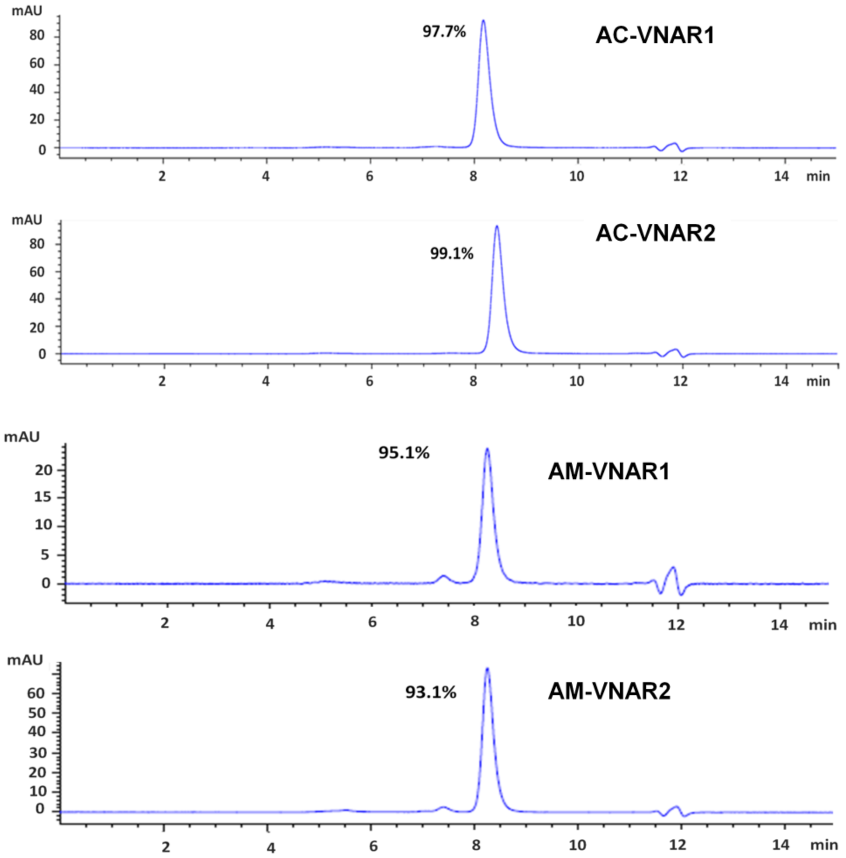

Figure S7: Chromatograms of analytical size exclusion chromatography (SEC) runs conducted with recombinantly expressed anti-ID vNARs as Fc fusions. Percentages indicate the purity of the monomeric Fc fusion. Analytical SEC runs were performed using a TSKgel SuperSW3000 column (4.6 x 300 mm, Tosoh) at a flow rate of 0.35 mL/min.

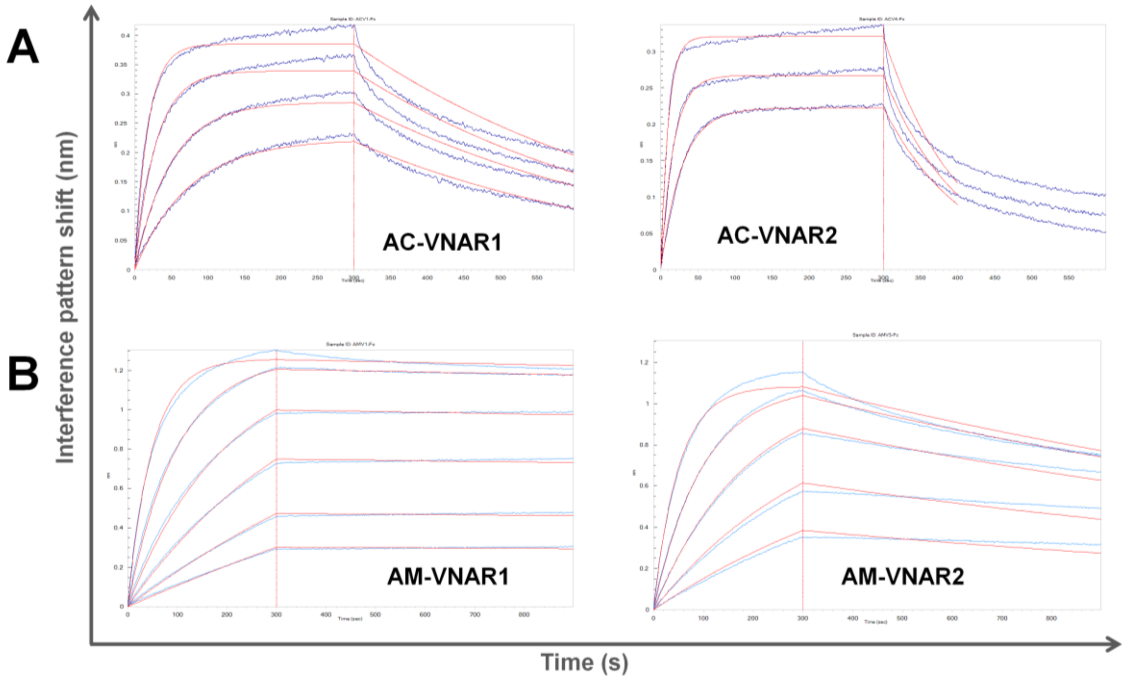

Figure S8: Binding kinetics of the anti-ID vNAR variants as determined using Bio-Layer Interferometry and an Octet® RED96 system. Sensorgrams were fitted using a 1:1 Langmuir binding model and global fitting of association and dissociation. cetuximab and matuzumab-biotin were immobilized onto anti-Fab-CH1 or streptavidin sensortips. After a baseline measurement in kinetics buffer for 120 s, association of decreasing concentrations of anti-ID vNAR-Fc variants was performed for 300 s. Dissociation was carried out in kinetics buffer for either 600 (AC-VNAR) or 900 s (AM-VNAR). A) Sensorgrams for kinetic analyses of AC-VNARs. B) Sensorgrams for kinetic analysis of AM-VNARs.

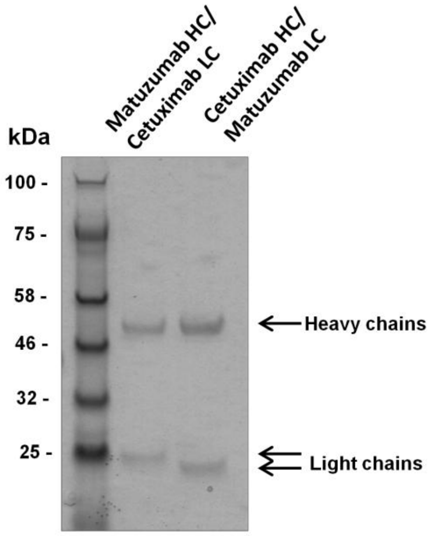

Figure S9: SDS-PAGE of mispaired cetuximab/matuzumab antibody variants. Both mispaired mAb constructs were recombinantly expressed in Expi293F^{TM} cells. The supernatant was subsequently purified using Protein A spin columns. As the SDS-PAGE comprises protein bands that correspond to heavy and light chains, successful assembly of the mispaired variants occured.
